# Transcriptional and translational analysis of subsequent infection with *Staphylococcus aureus* and *Proteus mirabilis* in *Caenorhabditis elegans*

**DOI:** 10.1186/1471-2334-14-S3-P7

**Published:** 2014-05-27

**Authors:** Udayakumar Prithika, Gnanasekaran JebaMercy, Krishnaswamy Balamurugan

**Affiliations:** 1 Department of Biotechnology, Alagappa University, Science Campus, Karaikudi-630004, India

## Background

*Caenorhabditis elegans* can be effectively used to study the dynamics of polymicrobial infections. *Proteus mirabilis* as an opportunistic pathogen does not cause death in *C. elegans*. Hence, in the present study, the *C. elegans* was pre infected with the pathogen *Staphylococcus aureus* to make the *C. elegans* immuno compromised to study the effect of *P. mirabilis* in the host.

## Methods

This study involved in investigation of impact of subsequent infections at physiological, transcriptional and translational levels using *C. elegans*.

## Results

Physiological status of *C. elegans* was analyzed using killing assay, CFU count and CLSM analysis. The mRNA expression analysis indicated the regulation of host specific immune regulatory genes, CUB like proteins (*F08G5.6*), neuropeptide like factors (*nlp-29*) and C type lectin genes (*clec-60* and *clec-87*) during subsequent infections. To study which protein is regulated against subsequent infections, SDS-PAGE analysis was performed using the proteins extracted from *C. elegans* exposed with subsequently infected samples for different time intervals. Results of 1D electrophoresis clearly suggested that subsequently infected samples showed significantly more differentially regulated proteins compared to their respective controls.

## Conclusion

The physiological assays and molecular studies demonstrated the vulnerability of a host as an integral event during *S. aureus* infections that enables the bacteria to suppress the host immune system, which subsequently lead to the opportunistic pathogen *P. mirabilis* to exert its pathogenicity in a host system. Further studies are needed to check the translational status of the host during subsequent infections.

